# Efficient and Easy Conversion of Human iPSCs into Functional Induced Microglia-like Cells

**DOI:** 10.3390/ijms23094526

**Published:** 2022-04-20

**Authors:** Jonas Lanfer, Johanna Kaindl, Laura Krumm, Miguel Gonzalez Acera, Markus Neurath, Martin Regensburger, Florian Krach, Beate Winner

**Affiliations:** 1 Department of Stem Cell Biology, Friedrich-Alexander-Universität Erlangen-Nürnberg (FAU), 91054 Erlangen, Germany; jonas.lanfer@fau.de (J.L.); johanna.kaindl@alumni.fau.de (J.K.); laura.krumm@uk-erlangen.de (L.K.); martin.regensburger@uk-erlangen.de (M.R.); flo.krach@fau.de (F.K.); 2 Department of Medicine 1, Friedrich-Alexander-Universität Erlangen-Nürnberg (FAU), 91054 Erlangen, Germany; miguel.gonzalezacera@uk-erlangen.de (M.G.A.); markus.neurath@uk-erlangen.de (M.N.); 3 Deutsches Zentrum Immuntherapie (DZI), 91054 Erlangen, Germany; 4 Center of Rare Diseases Erlangen (ZSEER), Friedrich-Alexander-Universität Erlangen-Nürnberg (FAU), 91054 Erlangen, Germany; 5 Department of Molecular Neurology, Friedrich-Alexander-Universität Erlangen-Nürnberg (FAU), 91054 Erlangen, Germany

**Keywords:** microglia, pluripotent stem cells, cell differentiation, neurology, immunology

## Abstract

Current protocols converting human induced pluripotent stem cells (iPSCs) into induced microglia-like cells (iMGL) are either dependent on overexpression of transcription factors or require substantial experience in stem-cell technologies. Here, we developed an easy-to-use two-step protocol to convert iPSCs into functional iMGL via: (1) highly efficient differentiation of hematopoietic progenitor cells (HPCs) from iPSCs, and (2) optimized maturation of HPCs to iMGL. A sequential harvesting approach led to an increased HPC yield. The protocol implemented a freezing step, thus allowing HPC biobanking and flexible timing of differentiation into iMGL. Our iMGL responded adequately to the inflammatory stimuli LPS, and iMGL RNAseq analysis matched those of other frequently used protocols. Comparing three different coating modalities, we increased the iMGL yield by culturing on uncoated glass surfaces, thereby retaining differentiation efficiency and functional hallmarks of iMGL. In summary, we provide a high-quality, easy-to-use protocol, rendering generation and functional studies on iMGL an accessible lab resource.

## 1. Introduction

Microglia represent the parenchymal tissue-resident macrophage population of the central nervous system (CNS). During development, microglia and other CNS-associated macrophages are derived from hematopoietic progenitor cells (HPCs) of the yolk sac which invade and further mature in the developing CNS [1].

Microglia exert crucial functions in neurodevelopment and CNS homeostasis including regulation of synaptic pruning and plasticity [2] and modulation of inflammation during CNS infection and other neurological disorders, including neurodegenerative diseases [3]. Thus, deciphering microglia functional states and understanding how interventions modulate their function hold a great promise to identify key mechanisms of CNS development, disease, and therapeutic interventions.

For a long time, microglia research relied on rodent systems, immortalized cell lines, or the availability of hardly accessible human brain tissue for isolation of primary human microglia. As rodent and human microglia harbor extensive differences in their transcriptome [4], the need to develop an easily accessible source for functional human microglia is apparent. Isolation of human embryonic stem cells (ESCs) or reprogramming of somatic cells to induced pluripotent stem cells (iPSCs) by overexpression of four transcription factors (OCT4, SOX2, KLF4, c-MYC) [5] have provided new valuable tools for research. In the past years, different protocols for the generation of human microglia-like cells (iMGL) from ESCs or iPSCs have been developed to overcome the issue of availability for human microglia [6,7,8,9,10,11,12,13]. However, current iMGL differentiation protocols still pose high infrastructural and technical boundaries, resulting in limited accessibility to a broad research community.

Two frequently used concepts of microglia generation are: (1) overexpression of specific transcription factors requiring lentiviral gene transfer in a Biosafety Level 2 (BSL2) certified lab [9] or extensive expertise in genomic engineering [14], and (2) differentiation using small molecules, recombinant proteins, and growth factors requiring extensive technical skills in differentiating progenitor cells from iPSC-derived embryonic bodies [7,8,11] and long periods for differentiation [6,8,10]. Notably, high variability in microglia cell yield, produced by those protocols, limits scalability.

To overcome these issues, we developed a simplified, scalable, and easily accessible two-step protocol for differentiation of microglia-like cells from iPSCs, which we term EZ-iMGL. We used a commercially available kit to generate hematopoietic progenitor cells (HPCs) and enhanced the protocol’s HPC yield by adding a sequential harvesting approach. Next, we explored and validated the option to cryopreserve HPCs, which adds temporal flexibility. These measures did not affect subsequent iMGL differentiation purity. Using RNAseq, we confirmed that the iMGL generated with our protocol exhibited a reliable and reproducible inflammatory response following LPS application, comparable to three other frequently used protocols. Lastly, we optimized the coating for iMGL maturation and delineated that maturing HPCs to iMGL on uncoated glass slides resulted in an increase of adherent iMGL that are capable of phagocytosis and responsive to LPS. Taken together, the protocol presented in this study provides an easily accessible and valuable resource to generate highly pure and functional microglia from iPSCs that substantially eases efforts to understand human microglia function in health and disease.

## 2. Results

### 2.1. Sequential Harvesting of HPCs Increases Yield of CD43+/CD45+ Microglia Progenitors

**Figure 1:**
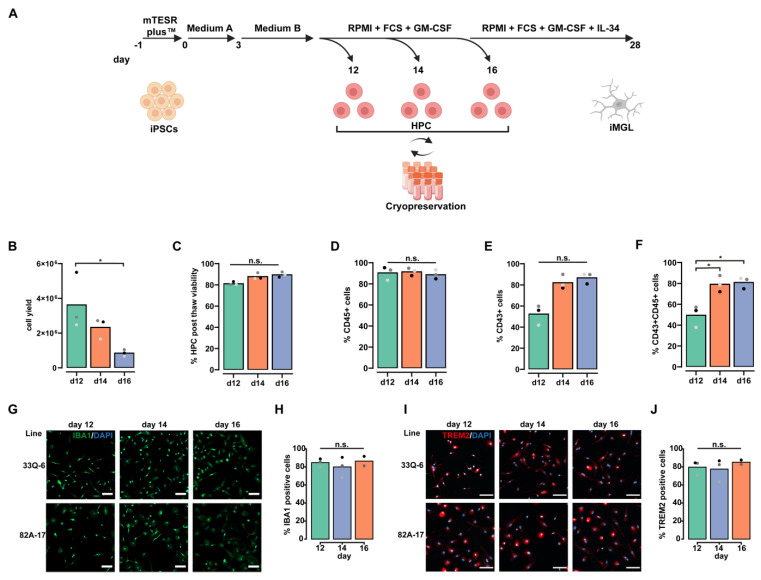
Sequential harvesting of HPCs increases yield of CD43+/CD45+ microglia progenitors. (**A**) Experimental paradigm depicting the iMGL differentiation protocol and highlighting the sequential harvesting and cryopreservation steps of HPC generation; (**B**) Bar plot depicting mean number of living cells present (bar) in the supernatant of cultures at day 12 (green), 14 (orange), or 16 (blue) during hematopoietic differentiation. Data are shown for *n* = 3 iPSC lines. Dots show individual values per iPSC line (black: 33Q-6, dark grey: 82A-17, light grey: CV-B). * depicts *p* = 0.0341 using One-way ANOVA with Tukey’s post hoc test; (**C**) Bar plot depicting mean viability (bar) after re-thawing of HPCs which were cryopreserved at day 12 (green), 14 (orange), and 16 (blue) of hematopoietic differentiation. Data are shown for *n* = 3 iPSC lines. Dots show individual values per iPSC line (black: 33Q-6, dark grey: 82A-17, light grey: CV-B). n.s. depicts *p* > 0.05 using Kruskal–Wallis test; (**D**) Bar plot depicting mean number of CD45 single positive cells (bar) in the supernatant of cultures at day 12 (green), 14 (orange), or 16 (blue) during hematopoietic differentiation as determined by FACS analysis. Data are shown for *n* = 3 iPSC lines. Dots show individual values per iPSC line (black: 33Q-6, dark grey: 82A-17, light grey: CV-B). n.s. depicts *p* > 0.05 using Kruskal–Wallis test; (**E**) Bar plot depicting mean number of CD43 single positive cells (bar) in the supernatant of cultures at day 12 (green), 14 (orange), or 16 (blue) during hematopoietic differentiation as determined by FACS analysis. Data are shown for *n* = 3 iPSC lines. Dots show individual values per iPSC line (black: 33Q-6, dark grey: 82A-17, light grey: CV-B). n.s. depicts *p* > 0.05 using Kruskal–Wallis test; (**F**) Bar plot depicting mean number of CD43/CD45 double positive cells (bar) in the supernatant of cultures at day 12 (green), 14 (orange), or 16 (blue) during hematopoietic differentiation as determined by FACS analysis. Data are shown for *n* = 3 iPSC lines. Dots show individual values per iPSC line (black: 33Q-6, dark grey: 82A-17, light grey: CV-B). * depicts *p* = 0.01 (day 12 vs. day 14) and *p* = 0.0076 (day 12 vs. day 16) using One-way ANOVA with Tukey’s post hoc test; (**G**) Representative fluorescence microscopy pictures depicting iMGL from iPSC lines 33Q-6 and 82A-17 were matured from HPCs harvested at respective days (12, 14, and 16) and stained for IBA1 (green) and DAPI (blue). Scale bar represents 100 µm; (**H**) Bar plot depicting mean percentage of IBA1 positive cells (bar) as quantified from fluorescence microscopy pictures at end of differentiation from HPCs which were harvested at day 12 (green), 14 (blue), and 16 (orange) and separately matured into iMGL. Data are shown for *n* = 3 iPSC lines for day 12 and 14 and *n* = 2 iPSC lines for day 16. Dots represent individual mean values per iPSC line (black: 33Q-6, dark grey: 82A-17, light grey: CV-B). n.s. depicts *p* > 0.05 using Kruskal–Wallis test; (**I**) Representative fluorescence microscopy pictures depicting iMGLs from iPSC lines 33Q-6 and 82A-17 were matured from HPCs harvested at respective days (12, 14, and 16) and stained for TREM2 (red) and DAPI (blue). Scale bar represents 100 µm; (**J**) Bar plot depicting mean percentage of TREM2 positive cells (bar) as quantified from fluorescence microscopy pictures at end of differentiation from HPCs which were harvested at day 12 (green), 14 (blue), and 16 (orange) and separately matured into iMGL. Data are shown for *n* = 3 iPSC lines for day 12 and 14 and *n* = 2 iPSC lines for day 16. Dots represent individual mean values per iPSC line (black: 33Q-6, dark grey: 82A-17, light grey: CV-B). n.s. depicts *p* > 0.05 using Kruskal–Wallis test.

To generate high quality and quantity HPCs reproducibly, we adapted the protocol of the STEMdiff™ Hematopoietic Kit (Stemcell Technologies) starting with small iPSC colonies (Figure S1A) using three iPSC-lines derived from fibroblasts of different healthy donors (Table S5). We established sequential harvesting steps at day 14 and 16 by including an HPC expansion medium and investigated the respective HPC quality and usability (Figure 1A). We collected and cryopreserved HPCs at day 12 (the end point of the kit), 14, and 16 of differentiation, and assessed HPC quality and differentiation capability after thawing. Across all iPSC lines, we observed the highest yields of harvested cells at day 12 and 14, with a decreasing yield at day 16 of HPC differentiation (Figure 1B). Notably, cryopreservation of the harvested HPCs did not affect their viability after thawing (Figure 1C). Next, we addressed the quality of our HPCs at day 12, 14, and 16 using flow cytometry analysis for the pan-hematopoietic marker CD45 and the leukocyte marker CD43. Throughout day 12, 14, and 16, ~90% of cells were positive for CD45 (Figure 1D). At day 12, half of the collected cells were single-positive for CD43 (Figure 1E) and double-positive for CD43/CD45 (Figure 1F). In contrast, we observed an increasing proportion of CD43+/CD45+ HPCs (Figure 1F and Figure S1B) at day 14 and 16, reflecting an increasing commitment to leukocyte identity over time in culture. To determine if the final differentiation into iMGL is affected by the day of HPC harvest, we matured the cells for 14 days and investigated the stained cells for the microglia/myeloid markers IBA1 and TREM2 (Figure 1G–J and Figure S1C,D). For all three HPC harvesting days, we observed around 80% of IBA1+ and TREM2+ microglia in the culture (Figure 1H,J), exhibiting typical ramified morphology (Figure 1G,I). This emphasizes that the sequential harvest and cryopreservation of HPCs leads to an increased cell yield without affecting differentiation capability. Since day 12 and day 14 HPC harvests led to the highest yield, we decided to pool and cryopreserve HPCs from those days for further experiments.

### 2.2. iMGL Elicit an Adequate LPS Response That Is Reproducible in other iMGL Protocols

**Figure 2:**
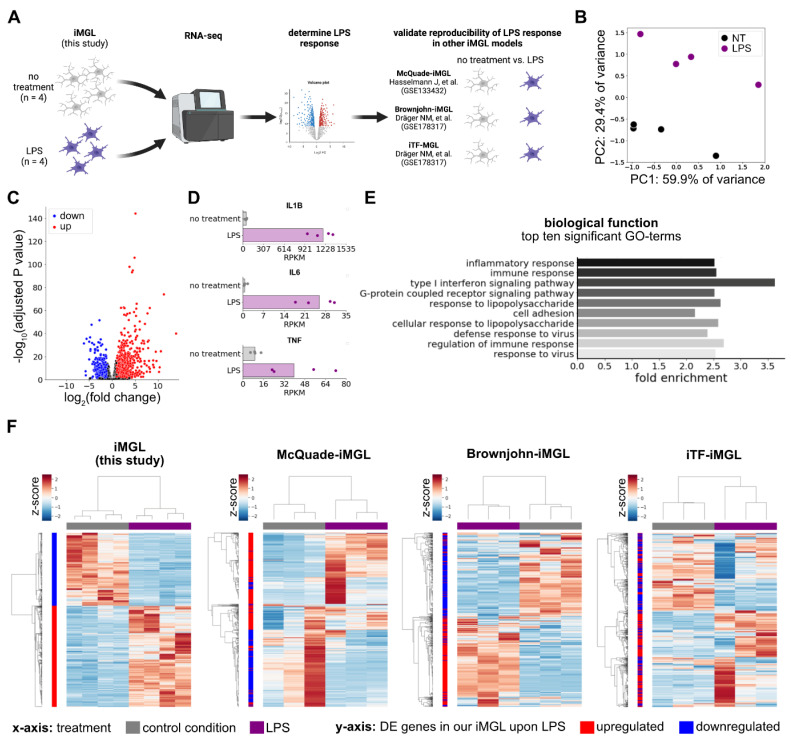
iMGL elicit an adequate LPS response that is reproducible in other iMGL protocols. (**A**) Experimental paradigm illustrating number of samples in our dataset (iPSC lines 33Q-6, 82A-17, CV-B) as well as publicly available datasets used from iMGL differentiated with other protocols; (**B**) PCA analysis presenting PC1 and PC2 of all RPKM values in our dataset with a mean RPKM ≥ 1 across all samples. NT = no treatment (black), LPS = lipopolysaccharide treatment (purple); (**C**) Volcano plot illustrating log_2_ (fold change) and negative log_10_ (adjusted *p* value) from DESeq2 output comparing LPS-treated iMGL with iMGL that were not subject to any treatment. Only genes with RPKM values in our dataset with a mean RPKM ≥ 1 across all samples were considered. red: adjusted *p* value ≤ 0.05, log_2_(fold change) ≥ 1; blue: adjusted *p* value ≤ 0.05, log_2_(fold change) ≤ 1; (**D**) Bar plot depicting median (bar) and individual (dots) RPKM values of IL1B, IL6, and TNF in our iMGL with and without LPS treatment; (**E**) Bar graph presenting fold enrichment values for the 10 most significant GO terms (Bonferroni corrected *p* value ≤ 9.385571 × 10^−5^) of DE genes upon LPS treatment in our iMGL compared to all genes with RPKM values in our dataset with a mean RPKM ≥ 1 across all sample; (**F**) Heatmaps with hierarchical clustering of samples and genes using correlation method of gene expression values of four different iMGL datasets containing samples with and without LPS treatment (from left to right: our dataset, McQuade-iMGL (GSE133432) [16], Brownjohn-iMGL (GSE178317), iTF-iMGL (GSE178317) [14]. Clustering was performed on LPS-responsive DE genes identified in (**C**). The heatmap illustrates z-scores of gene expression values from low (blues) to high (reds). The x-axis color bar represents treatment conditions: control conditions (gray) and LPS (purple). The y-axis color represents the change upon LPS in our iMGL dataset: upregulated (red) and downregulated (blue).

To investigate if the iMGL derived from sequentially harvested HPCs elicit a specific and reproducible inflammatory response in an unbiased manner, we differentiated HPCs into iMGL and performed RNA-seq at day 28 in untreated and LPS treated conditions followed by analysis of differential gene expression (DE) (Figure 2A, Table S1). Globally, reads per kilobase of transcript per million mapped reads (RPKM) values of genes expressed in the dataset (mean RPKM across all samples ≥ 1) were able to separate non-treated from LPS-treated samples in principal component analysis (PCA) (Figure 2B). Using DESeq2, a well-established algorithm for DE [15], we identified a total of 1486 genes as differentially expressed (adjusted *p* value ≤ 0.05, |log_2_(fold change)| ≥ 1), whereof the majority (862) were upregulated (Figure 2C, Table S1). Well-known LPS-responsive genes, such as IL1B, IL6 and TNF, could be identified among the upregulated genes (Figure 2D). Gene ontology (GO) analysis on all DE genes validated the specificity of the signal, computing high significance and enrichment of GO-term in the biological function category associated with inflammation, including ‘response to lipopolysaccharide’ (Figure 2E). In a last step, we asked whether the LPS response elicited by our iMGL is reproducible in iMGL generated with three previously published protocols. We included two LPS-treatment datasets of iMGL differentiated with small molecule-based protocols [14,16] and one dataset of iTF-iMGL, differentiated through overexpression of specific transcription factors (Figure 2A) [14]. We employed hierarchical clustering of LPS-responsive genes identified in our dataset (Figure 2C) to the respective expression values in the other three iMGL LPS datasets. Expectedly, using this analysis, LPS-treated samples were clearly separated in our iMGL (Figure 2F). Strikingly, a separation of non-treated vs. LPS-treated iMGL was also apparent for all three external datasets (Figure 2F). Interestingly, small molecule-based differentiation protocols (McQuade- and Brownjohn-iMGL) exhibited a very high concordance of the directionality of gene expression changes. This concordance was also detectable in iTF-iMGL, though to a lesser degree (Figure 2F). In conclusion, iMGL generated through our protocol elicited a specific response to inflammatory stimuli that can be reproduced in iMGL generated with previously published protocols.

### 2.3. Maturation on Glass Increases iMGL Yield While Retaining Purity and Functional Hallmarks of Microglia

**Figure 3:**
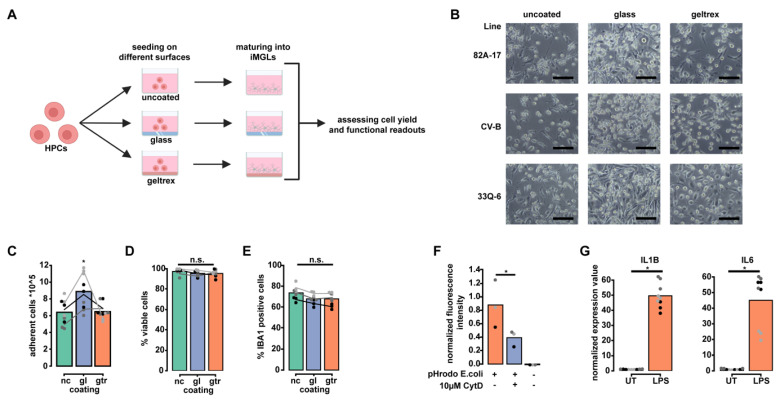
Maturation on glass increases iMGL yield while retaining purity and functional hallmarks of microglia. (**A**) Experimental paradigm depicting different coating modalities used for optimization of HPC maturation into iMGL; (**B**) Bright field pictures of iMGL differentiated from three different iPSC lines (33Q6, 82A-17 and CV-B) which were matured into iMGL on three different coating modalities (uncoated plastic, Geltrex™, glass). Scale bar represents 100 µm; (**C**) Bar plot depicting mean number of adherent cells (×10^5^, bar) as determined using cell counting at the end of differentiation. Data are shown for three replicates of *n* = 3 iPSC lines and three different coating modalities. nc = uncoated plastic (green), gl = glass (blue), gtr = Geltrex™ (orange). Dots represent individual values for three replicates of *n* = 3 iPSC lines (black: 33Q-6, dark grey: 82A-17, light grey: CV-B). Lines represent individual mean values for *n* = 3 iPSC lines (black: 33Q-6, dark grey: 82A-17, light grey: CV-B). * depicts *p*-value of *p* = 0.029 (glass vs. Geltrex™) and *p* = 0.0126 (uncoated vs. Geltrex™) using Kruskal–Wallis test with Dunn’s post hoc test; (**D**) Bar plot depicting mean percentage of viable cells (bar) at end of differentiation as determined using Live/Dead fluorescent cell counting after dissociation. Data are shown for three replicates of *n* = 3 iPSC lines and three different coating modalities. nc = uncoated plastic (green), gl = glass (blue), gtr = Geltrex™ (orange). Dots represent individual values for three replicates of *n* = 3 iPSC lines (black: 33Q-6, dark grey: 82A-17, light grey: CV-B). Lines represent individual mean values for *n* = 3 iPSC lines (black: 33Q-6, dark grey: 82A-17, light grey: CV-B). n.s. depicts *p* > 0.05 using Kruskal–Wallis test; (**E**) Bar plot depicting mean percentage of IBA1 (bar) at end of differentiation as determined by FACS analysis. Data are shown for three replicates of *n* = 3 iPSC lines and three different coating modalities. nc = uncoated plastic (green), gl = glass (blue), gtx = Geltrex™ (orange). Dots represent individual values for three replicates of *n* = 3 iPSC lines (black: 33Q-6, dark grey: 82A-17, light grey: CV-B). Lines represent individual mean values for *n* = 3 iPSC lines (black: 33Q-6, dark grey: 82A-17, light grey: CV-B). n.s. depicts *p* > 0.05 using One-way ANOVA; (**F**) Bar plot depicting mean fluorescence intensity normalized to nuclear signal (bars) of iMGL which were matured on glass slides and treated with pHrodo labelled *E. coli* bioparticles(orange), *E. coli* bioparticles plus 10 µM Cytochalasin D (blue), or left untreated (black). Data are shown for three replicates of *n* = 3 iPSC lines. Dots represent individual mean values for three replicates of *n* = 3 iPSC lines (black: 33Q-6, dark grey: 82A-17, light grey: CV-B). * depicts *p* = 0.0495 using Wilcoxon rank-sum test; (**G**) Bar plots depicting mean normalized expression values (bars) of iMGL matured on glass slides which were either left untreated or treated with LPS. Left diagram shows expression values for IL1B, right diagram shows expression values for IL6. Data are shown for three technical replicates of *n* = 3 iPSC lines. Dots represent individual values of *n* = 3 iPSC lines (black: 33Q-6, dark grey: 82A-17, light grey: CV-B). * depicts *p* = 0.0003 (IL1B untreated vs. LPS-treated) and *p* = 0.0003 (IL6 untreated vs. LPS-treated) using Wilcoxon rank-sum test.

A high yield and purity of differentiated cells enables an increasing number of applications such as biochemical fractionation of organelles and various proteomics approaches. To address this issue, we investigated if the iMGL yield after 2 weeks of maturation can be increased by culture on specific surfaces: uncoated plastic, as used in the previous experiments, a basement membrane matrix (here: Geltrex™) as used by others [6,10], and glass surfaces (Figure 3A). Culturing iMGL on glass resulted in an increased density of cells at the end of maturation compared to the other two conditions (Figure 3B and Figure S2A). This increase was also reflected in the cell yield after trypsin dissociation (Figure 3C). Notably, iMGL matured on Geltrex™ exhibited a more amoeboid morphology in contrast to the other conditions, indicative of an activated state (Figure 3B). Next, we assessed the purity of the three culture conditions and observed no significant differences in IBA1 positivity between the different coating modalities (Figure 3E and Figure S2B). Additionally, the viability was also unchanged (Figure 3D). To investigate if glass-cultured iMGL are functional, we measured phagocytosis of pHrodo-labeled bacterial particles and responsiveness to LPS as well-established functional readouts for iMGL function. We confirmed that the iMGL retained their phagocytic capabilities (Figure 3F) and responsiveness to LPS stimulation (Figure 3G). This highlights that culture on glass surfaces increases cell yield during maturation from HPCs into iMGL without affecting culture purity and retaining iMGL function.

## 3. Discussion

Here, we described a two-step protocol to generate high yield, functional iMGL with proper response to inflammatory stimuli. To emphasize the unique steps of our protocol, we advanced high quality HPC generation by adding a sequential harvest approach and included an optional cryopreservation step allowing the temporal separation of HPC generation. The matured iMGL exhibited a LPS response that can be replicated in iMGL differentiated with other popular differentiation protocols. Lastly, we optimized the yield of matured iMGL by comparing culture surfaces and identified glass as the ideal condition. By providing a detailed step-by-step technical description of the differentiation process (Supplementary File S1), this protocol will render research on human microglia more accessible.

While protocols for other stem cell-derived cell types such as neurons already exist for over a decade [5,17,18,19], publications on the generation of stem cell-derived microglia only emerged in recent years. From 2016 on, multiple protocols were provided by a variety of research groups [6,7,8,9,10,11,12,13]. During this timeframe, different conceptual approaches have emerged: small molecule-based differentiation protocols and overexpression of key microglia transcription factors. Small molecule-derived protocols usually aim to mimic the embryonic developmental trajectory in vitro, first generating HPCs and subsequent maturation into iMGL. Thus, the efficient and reliable differentiation into HPCs is the major bottleneck in small molecule-derived microglia differentiation procedures. Some published protocols make use of embryoid body (EB) driven mesoderm differentiation and subsequent harvesting of progenitor cells in the culture supernatant. However, a notable disadvantage of these protocols is the high amount of experimental effort and differentiation duration (>20 days) to generate homogeneous EB and HPCs [8,11]. In contrast, other protocols make use of an easier time-sparing monolayer approach for HPC differentiation using an optimized protocol, limiting the time and effort needed [6,10]. Our protocol adapts this approach by using a commercially available kit to generate HPCs. We refined these steps by inclusion of extended HPC harvest and cryopreservation, thereby increasing the HPC yield and smoothing out iPSC line-to-line variability. Similar to other protocols [8,10], maturing the HPCs to iMGL results in high purity culture with a reproducible LPS response. By maturing HPCs on glass surfaces, we increase the iMGL yield at the end of differentiation. Within the four-week timeframe, our adapted protocol results in an 80-fold increase from iPSCs to IBA1 positive iMGL on average, comparable to previously published small molecule-based protocols [10,13]. Importantly, due to the defined four-week differentiation period, we reduce required medium and costs to a minimum compared to long-term harvest approaches [7,13] while reaching reproducible results. 

In addition to small molecule-based approaches, there have been recent efforts to directly convert iPSCs into microglia via forced overexpression of key microglia transcription factors. A recently published protocol utilizes a lentivirus-mediated overexpression of the transcription factors SPI1 and CEBPA in iPSCs to generate functional microglia-like cells within less than 20 days [9]. However, only a small fraction (4.5% to 25%) of input iPSCs are converted into microglia-like cells. This cell loss during differentiation and the necessity to have access to a BSL2 lab limits the scalability and accessibility of this approach, especially regarding biomaterial-heavy applications. Another recently proposed protocol uses stable integration of an overexpression machinery for a larger set of microglia transcription factors into a safe harbor locus by CRISPR/Cas9 [14]. Although this approach is very elegant, the need for genome editing challenges experimental settings with large numbers of different iPSC lines.

## 4. Materials and Methods

### 4.1. iPSCs Cell Culture Maintenance

A total of three iPSCs-lines reprogrammed from fibroblasts of healthy control subjects were used for the experiments in this study (Table S5). Human iPSCs lines were cultured in mTeSR plus (STEMCELL Technologies, Vancouver, BC, Canada) at 37 °C with 5% CO_2_ on Geltrex™ (500 µg for 57 cm^2^, Thermo Fisher Scientific) coated plates. Cells were passaged as small clumps at a ratio of 1:3–1:10 every 3–5 days using Gentle Cell Dissociation Reagent (STEMCELL Technologies, Vancouver, BC, Canada) without any survival promoter, according to the manufacturer’s instructions. Regular testing for mycoplasma contaminations were performed using MycoAlert Mycoplasma Detection Kit (Lonza, Basel, Switzerland).

### 4.2. Differentiation of iPSCs into Microglia-like Cells

A detailed step-by-step protocol for generation of iMGL from iPSCs can be found in the supplementary materials (Supplementary File S1). In brief, human iPSCs were seeded as small clumps and differentiated into HPCs using the STEMDiff hematopoietic kit (STEMCELL Technologies Vancouver, BC, Canada) with the extension of an additional harvesting step on day 14 of culture using RPMI1640 + 10% FCS (Gibco, Waltham, MA, USA) + 10 ng/mL GM-CSF (Peprotech, Cranbury, NJ, USA) + Penicillin/Streptomycin which are not provided with the kit. HPCs harvested on day 12 and 14 were pooled and either cryopreserved or directly used for further maturation into iMGL. For maturation, HPCs were seeded in maturation medium (RPMI1640 + 10% FCS (Gibco, Waltham, MA, USA) + Penicillin/Streptomycin + 100 ng/mL IL-34 (Peprotech, Cranbury, NJ, USA) + 10 ng/mL GM-CSF (Peprotech, Cranbury, NJ, USA)) at a density of 90.000–100.000 cells/cm^2^ onto glass slides or the different coating modalities depicted in the respective experiments. Maturation medium was added every 2–3 days for 2 weeks with a half medium change after 1 week. After 2 weeks, iMGL were used for subsequent experiments.

### 4.3. Flow Cytometry (FC, FACS) Analysis of HPCs and iMGL

For FC analysis, cells were dissociated, collected and centrifuged at 300× *g* for 3 min and resuspended in FACS buffer (1× PBS + 2% FCS + 0.01% Sodium azide). A total of 100.000 cells per staining were transferred in a V-bottom 96 well plate. Cells were collected via centrifugation at 300× *g* for 3 min and fixed by incubation in 50 µL BD Cytofix/well (BD Biosciences, Franklin Lakes, NJ, USA) for 10 min at room temperature. After fixation, cells were permeabilized using 50 µL BD Perm/Wash (BD Biosciences Franklin Lakes, NJ, USA) for 5 min at room temperature followed by incubation with respective primary antibodies (Table S4) for 20 min at room temperature. After primary antibody incubation, cells were washed with BD Perm/Wash and either used directly for analysis or secondary antibody incubation for 20 min followed by another wash with BD Perm/Wash. After staining, cells were transferred to a fresh FACS tube for analysis on a Beckman Coulter Cytoflex S FACS platform. 

### 4.4. Immunofluorescence Stainings 

For immunofluorescence, HPCs were seeded for maturation into iMGL on coverslips at a density of 25,000 cells per cm^2^. After maturation, iMGL were fixed by incubation with 4% paraformaldehyde for 10 min at room temperature. After fixation, cells were blocked and permeabilized in blocking buffer (1× PBS, 5% Donkey Serum, 0.3% Triton-X-100) for 1 h at room temperature. After blocking, primary antibody was added in blocking solution followed by incubation for 1 h at room temperature. Cells were washed three times with washing buffer (1× PBS, 0.3% Triton-X-100) for 5 min followed by incubation with fluorescently labelled secondary antibodies for 1 h at room temperature. After incubation, DAPI was added to the cells (1:1000 in PBS) for 2 min and cells were washed three times in washing buffer before mounting on glass slides using Aqua/Poly-mount. Pictures were analyzed using CellProfiler (v4.0.7) and ImageJ software (v2.3.0) to count IBA1 or TREM2 positive cells per total cells as identified by DAPI staining. 

### 4.5. Phagocytosis Assay Using pHrodo Labeled E. coli Bioparticles 

For measurement of phagocytosis, iMGL were dissociated and 20,000 cells/96-Well were seeded in a fluorescence reader suitable 96-Well plate (Corning, Corning, NY, USA). After 48 h, pHrodo labelled *E. coli* bioparticles were added at a concentration of 200 ng/mL for 2 h at 37 °C. Cytochalasin D treated samples were pretreated with 10 µM Cytochalasin D for 30 min at 37 °C before addition of bioparticles. After 2 h, nuclei of cells were counterstained with NucBlue stain (Thermo Fisher, Waltham, MA, USA) for 20 min. After staining, fluorescence signal was measured on a Clariostar fluorescent plate reading device. 

### 4.6. Quantitative Real-Time-Time PCR 

For qPCR analysis, microglia-like cells were either treated with LPS (1000 EU/mL) or left untreated for 24 h. Cells were lysed in TriZOL reagent and RNA was extracted according to manufacturer’s instructions. A total of 500 ng RNA per sample was used for reverse transcription into cDNA using QuantiTect Reverse Transcripition Kit (Qiagen, Venlo, Netherlands) according to manufacturer’s instructions. PCR reactions were set up with the respective primers (Table S2) using SYBR Green mastermixes (Thermo Fisher, Waltham, MA, USA) or GAPDH TaqMan MasterMixes (Applied Biosystems, Waltham, MA, USA), according to the manufacturer’s instructions, and analyzed on a Roche LightCycler 480 real-time PCR platform. Ct values were normalized to three housekeeping genes (GAPDH, HPRT and B2M) and expression values relative to untreated samples were calculated using the ddCt method [20].

### 4.7. RNA Sequencing and Benchmarking of LPS Response

A total of 500 ng RNA per sample were sent to Genewiz Germany for RNA-seq for paired-end sequencing using a NovaSeq machine. We sequenced to a depth of ~20,000,000–30,000,000 reads. Resultant fastq files were aligned to hg38 using STAR version 2.7.0d [21] and assigned to genes using Subread’s featureCounts tool version 2.0.1 [22]. Reads Per Kilobase of transcript, per million mapped reads (RPKM) were calculated. Only genes with a mean RPKM value of ≥ 1 across the dataset were considered for further analysis. PCA analysis was performed on RPKM values using sklearn in Python 3 (v3.7.10). DESeq2 was used to determine differential gene expression [15]. Our significance cutoff was an adjusted *p* < 0.05 and |log_2_(fold change)| > 1. All downstream analysis was performed in Python 3 (v3.7.10) and graphs were generated using seaborn (v0.11.1). GO analysis of the biological function category was performed using DAVID [23]. All genes with a mean RPKM value of ≥1 across the dataset were used as a background. The correlation method was used for hierarchical clustering. We integrated gene expression values of publicly available datasets from non-treated and LPS-treated iMGL deposited on GEO: iMGL differentiated with the McQuade et al. protocol (GSE133432) [16], iMGL differentiated according to the Brownjohn et al. protocol (GSE178317), and iTF-iMGL generated via transcription factor overexpression (GSE178317) [14]. Available gene expression values of genes with differential gene expression upon LPS in our dataset were extracted from these other datasets and subject to hierarchical clustering, as explained above. Due to the Data Privacy Regulations covering individuals who donate biological material for iPSC generation, the generated RNA-seq data are only available through the corresponding author upon reasonable request.

### 4.8. Statistics

Graphs were generated using Graphpad Prism Software (GraphPad Software, San Diego, CA, USA) and the matplotlib (v3.3.4) and seaborn (v0.11.1) packages in Python 3 (v3.7.10). Statistical analysis and tests were performed using GraphPad Prism and the numpy (v1.19.2) scipy.stats (v1.6.1) and pandas (v1.2.3) packages in Python 3. Significance values and statistical tests used are depicted in the respective Figure legends.

## 5. Conclusions

In summary, we present an easy-to-use and scalable protocol for iMGL generation. To highlight the significance of our protocol, we boost the yield of HPC intermediates and differentiated iMGL by implementing a set of experimental measures. This protocol provides methodological advances and greatly simplifies the technical processes to provide human microglia research to a larger research community, thus opening new avenues for the efficient analysis of human microglia function in health and disease.

## Data Availability

The RNA-seq intermediate files (counts and DESeq2 output) are available in Table S1. Researchers can obtain access to lines and original files upon reasonable request to the corresponding author due to restrictions defined by the European Data Privacy Regulations.

## Supplementary Materials

Supplementary Materials can be downloaded at: https://www.mdpi.com/article/10.3390/ijms23094526/s1. Reference [24] is cited in Supplementary Materials.
