# Supplementary material for: Efficient and Easy Conversion of Human iPSCs into Functional Induced Microglia-like Cells
Source: Int J Mol Sci. 2022 Apr 20;23(9):4526. doi: 10.3390/ijms23094526 (PMC9105476; doi:10.3390/ijms23094526)
Supplement: Supplementary file 1 [file ijms-23-04526-s001.zip › Supplemental File S1.pdf]

Supplemental File S1 – EZ-iMGL: A Step-by-step protocol for generation of iMGL form iPSCs

Protocol at a glance

**Day**	**Instruction**
**-1**	seed iPSCs in small colonies
**0**	pick best splitting ratio, add 1ml Medium A
**2**	half medium change with Medium A
**3**	add 1ml Medium B
**5**	half medium change with Medium B
**7**	add 0.5ml Medium B
**10**	add 0.5ml Medium B
**12**	collect HPCs in collection medium
**14**	collect HPCs, freeze, seed
**15-21**	feed iMGL
**22**	half medium change
**23-27**	feed iMGL
**28**	Analyze iMGL or reseed

Media formulations

Medium	Compound	Stock	Dilution/volume
**mTESR plus iPS maintenance medium**	mTeSR Plus	1x	400ml
	mTeSR Plus supplement	5x	100ml
**Medium A**	STEMdiff hematopoietic basal Medium	1x	45 ml
	STEMdiff hematopoietic supplement A	200x	225µl
**Medium B**	STEMdiff hematopoietic basal Medium	1x	75ml
	STEMdiff hematopoietic supplement B	200x	375µl
**HPC collection medium**	RPMI 1640+ GlutaMax	1x	
	FCS	1x	
	PenStrep	100x	5ml
	GM-CSF	50µg/ml	1:5000 (end concenctration 10ng/ml) → add fresh before use
**Microglia differentation medium**	RPMI 1640 + GlutaMax	1x	450ml
	FCS	1x	50ml
	PenStrep	100x	5ml
	IL-34	100µg/ml	1:1000 (end concentration 100ng/ml) → add fresh before use
	GM-CSF	50µg/ml	1:5000 (end concentration 10ng/ml) → add fresh before use
**Freezing medium**	KOSR	1x	45ml
	DMSO (sterile)	10%	5ml

Cell culture compounds

Compound	Vendor	Catalog no.	stock
mTeSR Plus ES/iPS medium	Stemcell Techn.	05825	1x
RPMI1640 + GlutaMAX	ThermoFisher	61870036	1x
FBS, qualified and heat inactivated	Thermo	10270106	1x
Gentle Cell Dissociation Reagent			
IL-34, human recombinant	Peprotech	200-34	100µg/ml
GM-CSF, human recombinant	Peprotech	300-03	50µg/ml
Penicillin/Streptomycin	ThermoFisher	15140122	100x
STEMdiff Hematopoietic kit	Stemcell Techn.	05310	-
Geltrex LDEV free Matrix, hESC qualified	ThermoFisher	A1413302	9.6mg/ml, use at $0.02mg/cm^2$
DMSO, >99,5% BioScience grade	Roth	A994.1	1x
TrypLE Express	ThermoFisher	12605010	1x

Antibodies

Antibody	Vendor	Catalog no.	working dilution
rabbit anti IBA1	Wako	019-19741	FACS 1:50<br>IF 1:200
Donkey Anti-rabbit-AF 647	ThermoFisher	A-31573	FACS 1:200<br>IF 1:500
Donkey anti-rabbit AF-488	ThermoFisher	A-21206	FACS 1:200<br>IF 1:500
Anti CD-43-APC	Biolegend	343205	FACS: 1:50
Anti CD-45-PE-Cy7	Biolegend	103113	FACS: 1:50

Lab equipment

equipment	vendor
Axiovert 25 Light Microscope	Zeiss
SZX 10 Stereo Microscope	Olympus
Megafuge 16R centrifuge	Heraeus
Luna FL Dual Fluorescence Cell counter	Logos Biosystems

Step-by-step procedure

General remarks

It is important to start with high quality iPSCs grown on Geltrex in mTESR Plus without big areas of morphological differentiation. For this protocol, iPSCs which were around 60-90% confluent and have not been split at least two days before seeding were used at day -1. The protocol below is optimized for iMGL differentiation in a 12 well format using one 12 well plate per iPSC line. All volumes are depicted for a 12 well format. If needed, splitting ratios and volumes need to be scaled to other well formats. One 12 well plate of seeded iPSCs usually yields 5-20 million HPCs if differentiation is successful (see also Figure S1A)

Seeding iPSCs for differentiation (day -1)

1. At least 2 hours before seeding (alternatively day before seeding), coat three 12-well plates per line with Geltrex according to manufacturer's instructions.

2. Apirate medium from iPS cells and add 1ml of Gentle Cell Dissociation reagent to the cells.

3. Incubate cells for 7 minutes at room temperature.

4. In the meantime, aspirate the Geltrex/Matrigel solution from the freshly coated plates and add 1ml mTESR Plus to each well.

5. Aspirate the dissociation reagent and add 1ml mTESR Plus.

6. Scrape off the cells gently using a cell scraper (do not use any extensive force!).

7. To homogenize cell clumps into appropriate size, resuspend cells once using a 1000µl Pipette tip.

8. Seed the cells using splitting ratios of ~1:50 – 1:400 into the respective plates.

9. Incubate seeded iPSCs overnight in a 37°C incubator.

Troubleshooting: Make sure to swish the plate from time to time before aspirating the volume to be seeded as this avoids sinking of small iPSC clumps to the bottom of the plate.

Troubleshooting: Seeding at least three different splitting ratios is crucial in order to identify optimally seeded iPSCs before starting with HPC differentiation the day after.

Determining optimal seeding density (day 0)

This step is the crucial step of this protocol as the yield of HPC differentiation relies heavily on the cells not overgrowing. To guarantee a good HPC yield, we aim for around **16-40** small sized iPSC colonies in each well of the respective seeded 12 well plate. As clump size, attachment properties and cell viability after seeding is heterogeneous between each iPS line, seeding of 3 different splitting ratios per line is recommended. Figure S1A presents examples of iPSC-colonies of acceptable sizes and density at start of differentiation. Moreover, example pictures of oversized and undersized colonies are depicted. In order to determine the number of colonies in a given well, it may be helpful to use a Stereo-Microscope or any other suitable microscope with a low 1x or 0.8x magnification. As mentioned above, optimal colony number for a 12-well plate format is around 16-40 colonies per well.

10 After detection of the best splitting ratio for each line, discard the leftover plates.

11 Aspirate the medium on the chosen plates and add 1ml Medium A to each well.

12 Incubate the cells for 48 hours in a 37°C incubator.

Hematopoietic progenitor cell differentiation (day 2 – day 14)

Day 2

13 Aspirate half of the medium from each well and add 0.5ml fresh Medium A to each well.

14 Incubate the cells overnight in a 37°C incubator.

Day 3

15 Aspirate the medium from each well and add 1ml Medium B to each well.

16 Incubate cells for 48 hours in a 37°C incubator.

Note: At day 3, the colonies should begin to visibly grow in size and cells should start migrating outward from the colonies

Day 5

17 Aspirate half of the medium and add 0.5ml fresh Medium B to each well.

18 Incubate the cells for 48 hours in a 37°C incubator.

Day 7

19 Aspirate half of the medium and add 0.5ml fresh Medium B to each well

20 Incubate the cells for 72 hours in a 37°C incubator.

Day 10

Note: On day 10, colonies with migrating cells should have proliferated extensively. Moreover, colonies of bright round cells differentiating from the monolayer should have appeared. These cells represent hematopoietic progenitor cells (HPCs) that will increase in number during the next days of differentiation. Subsequently, these HPCs will lift off and will be collected in later steps of this protocol. To avoid loss of these cells, Medium B is only added to the respective wells on this day.

21 Add 0.5ml fresh Medium B to each well.

22 Prepare RPMI1640 medium + 10%FCS + P/S as a basal medium for HPC collection medium and iMGL differentiation medium.

23 Incubate the cells for 48 hours in a 37°C incubator.

Day 12 – 1st HPC collection

Note: At day 12 and 14, a lot of HPCs should be visible detaching from the adherent monolayer of cells into the supernatant of your culture.

24 Collect floating cells from each well by collecting the medium with a serological pipette into a 50ml conical tube. Avoid spraying medium on the adherent layer to prevent liftoff of adherent cells.

25 Add 1ml fresh collection medium to each well and put the plate back in a 37°C incubator.

26 Spin down floating cells at 500g for 5 minutes at room temperature.

27 Aspirate the supernatant and resuspend cells in 10ml fresh HPC collection medium.

28 Seed HPCs in a T75 cell culture flask and put them back in 37°C incubator.

29 Incubate the cells at 37°C for 48 hours.

Day 14 – 2nd HPC collection, freezing and seeding

2nd HPC collection

30 Collect floating cells from each well by collecting the medium with a serological pipette into a 50ml conical tube. Avoid spraying medium on the adherent layer to prevent liftoff of adherent cells.

31 Combine the HPC containing supernatant of day 14 with the supernatant from day 12, mix well by pipetting up and down with a serological pipette

32 Determine the concentration of cells in the supernatant and calculate the number of total cells in your culture.

Note: In this protocol, we used a dual fluorescence Luna cell counter with a propidium-iodide stain and a lower counting cutoff of 6µm. However, any other suitable method to count cells like a Neubauer chamber may also be used for this step.

33 Freeze down collected HPCs or seed directly for further differentiation

(Optional) Freezing HPCs

i. To freeze HPCs, spin down the desired number of cells at 500g for 5 mins.

ii. Aspirate medium and resuspend cells in an appropriate volume of freezing medium.

Note: For this protocol, working aliquots of 2 Mio HPCs at a concentration of 1 Mio cells/ml have been proven useful. However, depending on the amount of cells, also other aliquot sizes may be used.

iii. Quickly add the cell suspension into cryovials and put them in a Mr. Frosty/Cell Camper at -80°C.

iv. After a minimum of 2hrs – overnight, store frozen vials in liquid nitrogen.

Note: It is not recommended to store HPCs at -80°C for an extended period of time.

Seeding HPCs for further differentiation

(Optional) Thawing cryopreserved HPCs

i. To thaw cryopreserved HPCs, thaw desired number of cryovials in a 37°C water bath for 1-2 minutes.

ii. Extensively disinfect the vials using 70% Ethanol to prevent contamination.

iii. Transfer the cell suspension into a 50ml conical tube

iv. Wash the cell suspension once by adding 5x the amount of 37°C warm RPMI 1640 + 10% FCS.

v. Spin down cells at 500g for 5 min at room temperature.

vi. Aspirate the supernatant and resuspend cells in fresh microglia differentiation medium.

34 Spin down desired amount of HPCs to be seeded at 500g for 5 min at room temperature.

35 Resuspend HPCs in fresh Microglia differentiation medium

36 Determine the cell concentration using a suitable cell counting method (also see Step 32 for details on counting of HPCs)

37 Seed cells at desired density onto glass bottomed cultureware

Quality control – FACS analysis of CD43 and CD45 on HPCs

Note: This protocol should yield a highly pure population of HPCs with around 90% of cells expressing the pan-hematopoietic marker CD45 and >50% of cells expressing the leukocyte marker CD43 as determined by FACS. The workflow for FACS analysis of HPCs is as follows:

i. Collect appropriate amount of supernatant containing HPCs and spin cells down at 300g for 3 mins
ii. Resuspend HPCs in FACS buffer (1x PBS + 2%FCS + 0.01% Sodium azide) and count cell concentration (see step 32 for details on counting)
iii. Add 100.000 cells per staining to a V-bottom 96 well plate.
iv. Spin down cells again at 300g for 3 minutes

v. Optional (if fixation of cells is required): Aspirate supernatant and add 50µl BD Cytofix/well (BD Biosciences) for 10 minutes at room temperature.
vi. Optional (if fixation of cells is required): Spin down cells at 300g for 3 minutes
vii. Aspirate supernatant and add primary antibody solution in FACS buffer (see Antibody Table for details)
viii. Incubate cells for 20 minutes at room temperature
ix. Spin down cells at 300g for 3 minutes and wash cells by resuspension in 50µl BD Perm/Wash
x. Spin down cells at 300g for 3 minutes and reuspend cells in 100µl fresh FACS buffer, transfer solution into fresh FACS tube
xi. Analyze cells on an appropriate Flow Cytometry platform

Note: For optimal adherence of iMGL and maximum cell yield, it is beneficial to seed HPCs onto glass-bottomed plates (e.g. chamber slides or 12/24well plates containing coverslips) Usual numbers to seed are 400k cells per 12 well for downstream analysis such as qPCR or harvesting for FACS analysis or 25k-50k cells per 24 well for microscopy. For Western Blot, pooling of two 12 wells is recommended. There is no need for coating of the desired culture vessel. It is recommended to differentiate HPCs in the respective analysis plates/vessels as reseeding of differentiated iMGL is time consuming.

Microglia maturation (day 14 – day 28)

Day 15 – Day 21 – feeding iMGL

Note: During the first week of differentiation, most cells will still be floating and not grow adherent . Moreover, HPCs tend to proliferate a little in this timeframe. It is important to add fresh growth factors to the medium right before each use. After one week, a half medium change will be performed.

38 Feed differentiating iMGL by adding 0.5ml fresh microglia differentiation medium every other day.

Day 22 – half medium change

39 Collect non-adherent cells in a 50ml conical tube leaving 0.5ml of medium in the well.

40 Put the plate back in the 37°C incubator.

41 Spin down the floating cell suspension at 500g for 5mins at room temperature.

42 Aspirate the supernatant and resuspend the cells in 0.5ml fresh microglia differentiation medium per well.

43 Add 0.5ml of resuspended floating cell suspension per well back into the plate

Day 23-28 - feeding iMGL

44 Feed iMGL every other day by adding 0.5ml fresh microglia differentiation medium.

Day 28 – Analysis and optional reseeding

At day 28, iMGL with a typical ramified morphology should adhere to the cultureware.

(Optional) Reseeding or harvesting iMGL

i. Warm Trypsin to 37°C in a water bath.

ii. Aspirate medium from respective wells and wash cells once with room temperature PBS w/o Calcium and Magnesium.

iii. Add 0.5ml of warm Trypsin to each well and incubate for 20 minutes at 37°C.

iv. After 20 minutes, add 0.5ml RPMI 1640 + 10%FCS to stop the trypsin reaction.

v. Gently scrape off the cells using a cell scraper and collect them in a conical tube.

vi. Spin down cells at 500g for 5 mins at room temperature.

vii. Resuspend iMGL in desired amount of fresh microglia differentiation medium and count the cells using an appropriate method.

Note: For this protocol, the same counting conditions as for HPCs were used (also see step 32 for more details on the counting procedure).

viii. Reseed iMGL at desired density needed for downstream analysis (See day 28 for more information on appropriate cell densities for downstream applications)

Quality control – Determining IBA1 positivity using FACS

Note: This protocol should yield a highly pure iMGL population with around 70-80% of cells expressing the microglial/myeloid marker IBA1 as determined by FACS analysis. The workflow for FACS analysis of iMGL is as follows:

i. Collect appropriate amount of iMGL by harvesting with Trypsin (see section above for details).
ii. Resuspend iMGL in FACS buffer (1x PBS + 2%FCS + 0.01% Sodium azide) and count cell concentration (see step 32 for details on counting)
iii. Add 100.000 cells per staining to a V-bottom 96 well plate.
iv. Spin down cells at 300g for 3 minutes
v. Aspirate supernatant and add 50µl BD Cytofix/well (BD Biosciences) for 10 minutes at room temperature for fixation of cells.
vi. Spin down cells at 300g for 3 minutes
vii. Aspirate supernatant and add 50µl permeabilization buffer (1xBD Perm/Wash (BD Biosciences) + 0.05% Triton-X-100 in PBS) for permeabilization of cells
viii. Incubate for 5 minutes at room temperature
ix. Spin down cells at 300g for 3 minutes
x. Aspirate supernatant and add primary IBA1 antibody solution in permeabilization buffer (see Antibody table for details)
xi. Incubate cells for 20 minutes at room temperature
xii. Spin down cells at 300g for 3 minutes and wash cells by resuspension in 100µl permeabilization buffer
xiii. Spin down cells at 300g for 3 minutes, aspirate supernatant and secondary antibody solution in permeabilization buffer (see Antibody table for details)

xiv. Spin down cells at 300g for 3 minutes and wash cells by resuspension in 100µl permeabilization buffer

xv. Spin down cells at 300g for 3 minutes and resuspend cells in 100µl fresh FACS buffer, transfer solution into fresh FACS tube

xvi. Analyze cells on an appropriate Flow Cytometry platform
