# Supplementary material for: Efficient and Easy Conversion of Human iPSCs into Functional Induced Microglia-like Cells
Source: Int J Mol Sci. 2022 Apr 20;23(9):4526. doi: 10.3390/ijms23094526 (PMC9105476; doi:10.3390/ijms23094526)
Supplement: Supplementary file 1 [file ijms-23-04526-s001.zip › Supplemental Information.pdf]

Supplemental Information

Figure S1 (related to Figure 1 and Supplemental File 1)

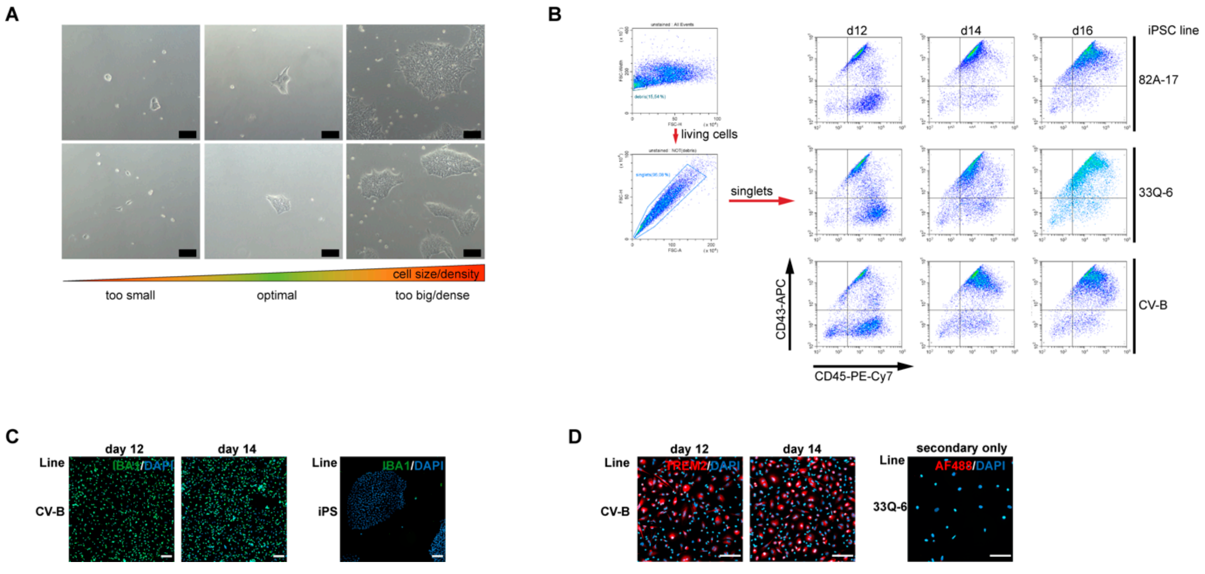

Figure S1 (related to Figure 1 and Supplemental File S1)
(A) Representative bright field pictures of iPSCs at start of HPC differentiation. Depicted are two examples of too small, optimally seeded and too big/dense iPSC colonies. Scale bar represents 100µm. (B) Scatterplots depicting gating strategy for FACS analysis of CD43/CD45 on HPCs. Scatterplots depicting gating of living cells and singlets (two left scatterplots). The grid on the right half shows scatterplots of CD43 fluorescence intensity (y-axis) vs. CD45 fluorescence intensity (x-axis) for HPCs differentiated from 3 different iPSC lines (82A-17, 33Q-6, CV-B) that were harvested on day 12, 14 and 16 and depicts the gates which were used to identify CD43+, CD45+ and CD43/CD45 double positive cells in subsequent analysis. (C) Fluorescence microscopy pictures depicting iMGLs from iPS line CV-B which were matured from HPCs harvested at respective days (12 and 14) and stained for IBA1 and DAPI (two left panels). Fluorescence microscopy pictures of undifferentiated iPS cells stained for IBA1 and DAPI as negative control (right panel). Scale bar represents 100µm. (D) Fluorescence microscopy pictures depicting iMGLs from iPS line CV-B which were matured from HPCs harvested at respective days (12 and 14) and stained for TREM2 (red) and DAPI (blue) (two left panels). Fluorescence microscopy picture of iMGLs differentiated from iPS line 33Q-6 stained with Alexa-Fluor 488 labelled secondary antibody only and DAPI as negative control (right panel). Scale bar represents 100µm.

Figure S2 (related to Figure 3)

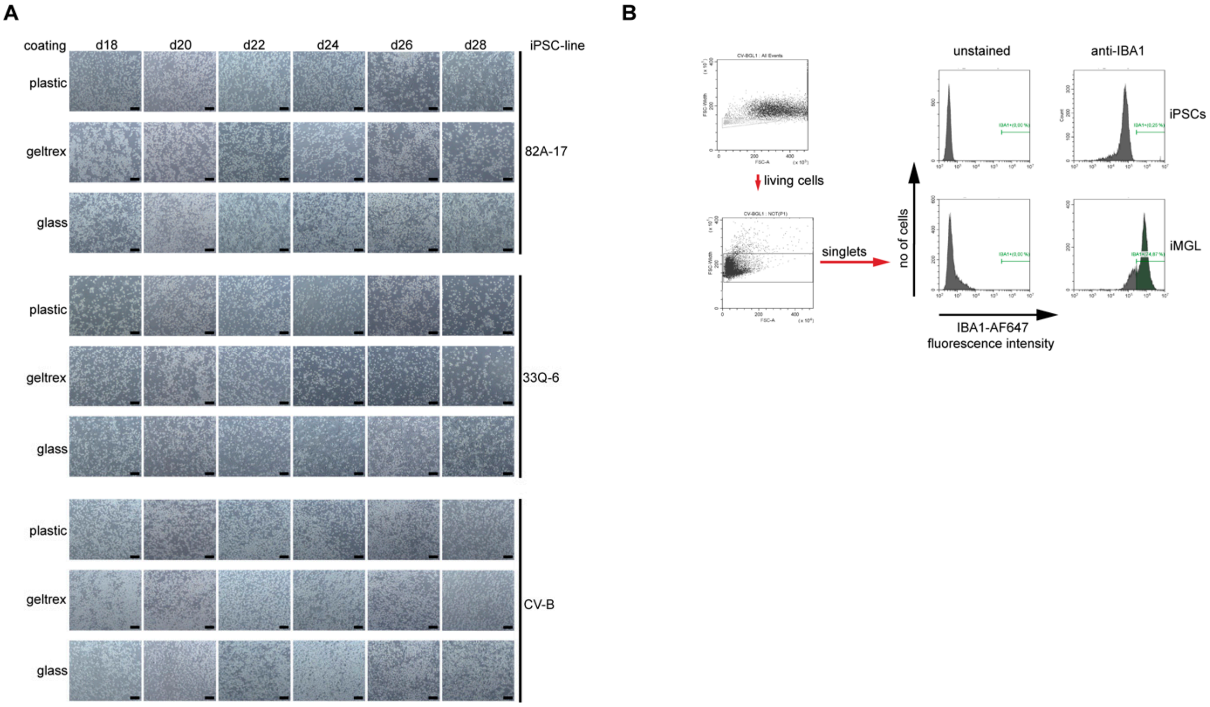

Figure S2 (related to Figure 3)
(A) Representative bright field pictures of HPCs matured into microglia on three different coatings (plastic, geltrex and glass) over the course of maturation from day 18 to day 28. Depicted are HPCs from three different iPSC lines (82A-17, 33Q-6, CV-B) that were further matured into iMGL. Scale bar represents 100µm. (B) Gating strategy for FACS analysis of IBA1 positivity on iMGL. Scatterplots depicting gating of living cells and singlets (two left scatterplots). The right half of the figure shows 4 histograms depicting no of cells (y-axis) vs. fluorescence intensity on unstained and anti-IBA1 stained iPSCs serving as negative control and iMGL differentiated from one iPSC line (CV-B) which were either left unstained or stained with anti-IBA1 antibody. The histograms include the gate which was used for analysis of IBA1-positive cells in subsequent analysis.

Tables

Table S1 – RNAseq intermediate files (provided as file)

Table S2 – qPCR primers

Target mRNA	Fw primer	Rev primer
IL1B	TCCAGGGACAGGATATGGAG	CCCAAGGCCACAGGTATTT
IL6	AGACAGCCACTCACCTCTTCAG	TTCTGCCAGTGCCTCTTTGCTG
GAPDH	GAPDH TaqMan Mastermix (Applied Biosystems #402869)	GAPDH TaqMan Mastermix (Applied Biosystems #402869)
HPRT	CCTGGCGTCGTGATTAGTG	TCCCATCTCCTTCATCACATC
B2M	GAGGCTATCCAGCGTACTCC	AATGTCGGATGGATGAAACC

Table S3 – Cell culture compounds

Compound	Vendor	Catalog no.	stock
mTeSR Plus ES/iPS medium	Stemcell Techn.	05825	1x
RPMI1640 + GlutaMAX	ThermoFisher	61870036	1x
FBS, qualified and heat inactivated	ThermoFisher	10270106	1x
Gentle Cell Dissociation Reagent	StemCell Technologies	100-0485	1x
IL-34, human recombinant	Peprotech	200-34	100µg/ml
GM-CSF, human recombinant	Peprotech	300-03	50µg/ml
Penicillin/Streptomycin	ThermoFisher	15140122	100x
STEMdiff Hematopoietic kit	StemCell Technologies	05310	-
Geltrex LDEV free Matrix, hESC qualified	ThermoFisher	A1413302	9.6mg/ml, use at 0.02mg/cm^2
DMSO, >99,5% BioScience grade	Roth	A994.1	1x
TrypLE Express	ThermoFisher	12605010	1x

Table S4- Antibodies

Antibody	Vendor	Catalog no.	working dilution
rabbit anti IBA1	Wako	019-19741	FACS 1:50<br>IF 1:100-1:200
goat anti TREM2	R+D systems	AF1828	IF 1:200
donkey anti rabbit AF 647	ThermoFisher	A-31573	FACS 1:200<br>IF 1:500
donkey anti rabbit AF 488	ThermoFisher	A-21206	FACS 1:200<br>IF 1:500
donkey anti goat AF488	ThermoFisher	A-11055	IF 1:500
anti CD43 APC	Biolegend	343205	FACS: 1:50
anti CD45 PE-Cy7	Biolegend	103113	FACS: 1:50

Table S5 – iPSC lines used in this study

Cell line	Code	Age at donation	Gender	origin
33Q-6	UKERi-33Q-R2-006	45	female	University Hospital Erlangen
82A-17	UKERi-82A-S1-017	66	female	University Hospital Erlangen
CV-B	CV-hiPS-B	63	male	Gore et al., 2011 [23]

Files

Supplemental File S1 – Step-by-step protocol
